# Radiomics signature for prediction of lateral lymph node metastasis in conventional papillary thyroid carcinoma

**DOI:** 10.1371/journal.pone.0227315

**Published:** 2020-01-15

**Authors:** Vivian Y. Park, Kyunghwa Han, Hye Jung Kim, Eunjung Lee, Ji Hyun Youk, Eun-Kyung Kim, Hee Jung Moon, Jung Hyun Yoon, Jin Young Kwak

**Affiliations:** 1 Department of Radiology, Severance Hospital, Research Institute of Radiological Science, Yonsei University College of Medicine, Seoul, Korea; 2 Department of Radiology, Kyungpook National University Chilgok Hospital, School of Medicine, Kyungpook National University, Daegu, Korea; 3 Department of Computational Science and Engineering, Yonsei University, Seoul, Korea; 4 Department of Radiology, Gangnam Severance Hospital, Research Institute of Radiological Science, Yonsei University College of Medicine, Seoul, Korea; Mayo Clinic College of Medicine, UNITED STATES

## Abstract

**Purpose:**

Preoperative neck ultrasound (US) for lateral cervical lymph nodes is recommended for all patients undergoing thyroidectomy for thyroid malignancy, but it is operator dependent. We aimed to develop a radiomics signature using US images of the primary tumor to preoperatively predict lateral lymph node metastasis (LNM) in patients with conventional papillary thyroid carcinoma (cPTC).

**Methods:**

Four hundred consecutive cPTC patients from January 2004 to February 2006 were enrolled as the training cohort, and 368 consecutive cPTC patients from March 2006 to February 2007 served as the validation cohort. A radiomics signature, which consisted of 14 selected features, was generated by the least absolute shrinkage and selection operator (LASSO) regression model in the training cohort. The discriminating performance of the radiomics signature was assessed in the validation cohort with the area under the receiver operating characteristic curve (AUC).

**Results:**

The radiomics signature was significantly associated with lateral cervical lymph node status (*p* < 0.001). The AUC of its performance in discriminating metastatic and non-metastatic lateral cervical lymph nodes was 0.710 (95% CI: 0.649–0.770) in the training cohort and was 0.621 (95% CI: 0.560–0.682) in the validation cohort.

**Conclusions:**

The present study showed that US radiomic features of the primary tumor were associated with lateral cervical lymph node status. Although their discriminatory performance was slightly lower in the validation cohort, our study shows that US radiomic features of the primary tumor alone have the potential to predict lateral LNM.

## Introduction

Papillary thyroid carcinoma (PTC) is the most common histologic type of thyroid malignancy, and most patients have a favorable prognosis [1]. PTC primarily accounts for the increased incidence rates of thyroid cancer seen in the past several decades, which is mainly due to the early detection of small tumors with high-resolution ultrasonography (US) [2,3]. Subsequently, overall mortality of thyroid cancer has also significantly decreased, with five-year cancer-specific mortality rates decreasing from 5.9% to 0.2% during the last four decades in South Korea [3].

However, approximately 13.3% of patients experience recurrence after initial surgery, with most PTC recurrences occurring in the cervical lymph nodes [3,4]. Although PTC nodal metastases have little influence on survival, they have been well associated with increased risk of recurrence and potentially associated with mortality in select patient populations [5,6]. Patients with pathologic lateral lymph node metastasis (LNM) typically require aggressive treatment, including lateral compartment lymph node dissection and high-dose radioactive iodine (RAI) therapy [7]. In addition, lateral LNM conveys a higher risk of recurrence than central LNM, even when treated with therapeutic neck dissection and/or RAI ablation, which increases morbidity and reduces quality of life [5,8]. Therefore, identification of lateral LNM is crucial for establishing appropriate management strategies. Preoperative neck US and US-guided fine-needle aspiration biopsy are the primary tools used for detecting and diagnosing LNM in patients with thyroid cancer [9]. However, the diagnostic performance of preoperative neck US differs among physicians, and interobserver variability is highest when determining lateral LNM [10,11].

Fueled by the exponential growth of medical imaging and developments in analytical methods, the field of radiomics has attracted increased attention in recent years. Previous studies have shown that quantitative radiomics features can provide insight into personalized medicine and potentially improve diagnostic, prognostic, and predictive accuracy [12]. Radiomics signatures, which consist of radiomic features, have been associated with clinical prognosis across a wide range of cancer types and are conveniently used to facilitate the preoperative individualized prediction of LNM [13–15]. However, there are only a few studies investigating the association of US-based radiomic features with LNM in patients with PTC, and fewer or no studies specifically focusing on lateral LNM [16,17].

Thus, in this study we aimed to develop a radiomics signature using US images to preoperatively predict lateral LNM in patients with conventional PTC (cPTC).

## Materials and methods

### Patients

The Severance Hospital Institutional Review Board approved this retrospective study and waived the need to obtain informed consent (Approval number: 4-2017-1024). All research was performed in accordance with relevant guidelines. The training cohort of this study was consecutive patients with histologically confirmed cPTC who underwent preoperative US and thyroid surgery from January 2004 to February 2006, and who did not have evidence of distant metastases at diagnosis. In total, 400 patients were identified and comprised the training cohort (342 women and 58 men; mean age, 45.37 years ± 12.90; range, 17 to 80 years). An independent validation cohort was obtained from 368 consecutive patients (306 women and 62 men; mean age, 44.92 years ± 12.26; range, 18 to 76 years) with surgically confirmed cPTC from March 2006 to February 2007, using the same criteria as the training cohort.

Total or near-total thyroidectomy was performed in patients either diagnosed or suspected of having multiple tumors, extrathyroidal extension or LNM upon preoperative evaluation or intraoperative findings. All patients underwent routine central compartment neck dissection, including the pretracheal, paratracheal, and prelaryngeal lymph nodes. Bilateral or ipsilateral central compartment neck dissection was performed in patients undergoing total or near-total thyroidectomy or in those undergoing hemithyroidectomy, respectively. In patients diagnosed with lateral LNM by preoperative US-guided fine-needle aspiration, lateral compartment neck dissection was performed. Intraoperative frozen biopsy was also performed for lymph nodes suspicious for metastases found during surgery which were not found on preoperative US. If lateral LNM was confirmed, lateral compartments including levels 2, 3, 4 and anterior 5 were dissected. Clinicopathologic data were collected by reviewing medical records.

### US image acquisition and region-of-interest segmentation

All patients underwent preoperative US of both thyroid glands and the cervical regions, performed by using a 5−13-MHz (SONOLINE Antares; Siemens Medical Solutions, Erlangen, Germany/ Acuson Sequoia 512; Acuson, Mountain View, CA), a 7−12-MHz (HDI 3000 or 5000; Philips Medical Systems, Bothell, Wash), or a 5−12-MHz linear array transducer (iU22; Philips Medical Systems, Bothell, Wash). For feature selection, a representative US image was selected for each tumor. Representative US images were selected from images that were previously captured by a radiologist at the time of the examination, which were retrieved from the picture archiving and communication system.

All manual segmentations of the thyroid tumors were performed by a radiologist (V.Y.P.) who had 9 years of experience in thyroid US imaging by delineating a region of interest (ROI) around the boundary of the index tumor on the representative US image. Each segmentation was validated by a senior radiologist (K.J.Y.) who had 18 years of subspecialty experience in thyroid imaging.

### Radiomic feature extraction, selection and building the radiomics signature

Texture feature extraction was performed by in-house texture analysis algorithms implemented in MATLAB 2016b (The MathWorks, Inc., Natick, Massachusetts, United States). The ROI-segmented US images were saved as JPG images and then converted into grayscale intensity images by eliminating the hue and saturation information while retaining luminance. In total, 730 candidate radiomic features, including energy, entropy, kurtosis, features using GLCM and GLRLM texture matrices, features using single-level discrete 2D wavelet transform and so on, were generated from a single US image. Each extracted ROI image was normalized for direct comparison between patients when textural features were calculated. More information about the methodology for radiomic feature extraction is described in the S1 Appendix.

We used the least absolute shrinkage and selection operator (LASSO) method in logistic regression to select the most useful predictive radiomic features for lateral cervical LNM from the training cohort. The statistical analyses were performed using R software, version 3.3.3 (http://www.R-project.org), where the package ‘glmnet’ was used to apply the LASSO method. The LASSO method is a penalized technique for variable selection that is suitable for the regression of high-dimensional data[18]. In the LASSO logistic regression analysis, 10-fold cross-validation was used to avoid overfitting. The selected imaging features were then combined into a radiomics signature. Based on the estimated coefficients, we computed a radiomics score (Rad-score) for each patient to reflect the risk of lateral LN metastasis. The predictive accuracy of the radiomics signature was calculated by using the area under the receiver operating characteristic curve (AUC) in both the training and validation cohorts.

### Statistical analysis

The statistical analyses were performed using R software, version 3.3.3 (http://www.R-project.org), where the package ‘glmnet’ was used to apply the LASSO method. All other statistical tests were conducted using the basic R functions. Differences in lateral LNM prevalence and clinicopathologic characteristics between the training and validation cohort were assessed with the Mann-Whitney *U* test and the Chi-Square test. A two-sided *P* < 0.05 was considered statistically significant.

## Results

### Patient characteristics and thyroid nodules

The clinical characteristics of the training and validation cohorts are given in Table 1. The training cohort had a larger pathological tumor size than the validation cohort, but the difference was only 1.2 mm (19.27 mm vs. 18.02 mm, *p* = 0.007). The validation cohort had a slightly higher lateral LNM prevalence than the training cohort (21% vs. 27%, *p* = 0.045). No significant differences were found between the two cohorts in terms of age (*p* = 0.763) and sex (*p* = 0.426). We also compared the above basic information between patients with lateral LNM and without lateral LNM in the training and validation cohorts, respectively. Details are shown in Table 2.

**Table 1:** Patient and tumor characteristics in the training and validation cohorts.

	Training cohort (n = 400)	Validation cohort (n = 368)	*p-*value
Age (years) ^a^	45 (35, 54)	46 (35, 53)	0.763
Sex			0.426
Female	342 (86.0%)	306 (83.0%)	
Male	58 (14.0%)	62 (17.0%)	
Pathological tumor size (mm) *	17 (13, 23)	15 (12, 22)	0.007
Central lymph node metastasis			0.725
Negative	170 (42.5%)	162 (44.0%)	
Positive	230 (57.5%)	206 (56.0%)	
Lateral lymph node metastasis			0.045
Negative	317 (79.0%)	268 (73.0%)	
Positive	83 (21.0%)	100 (27.0%)	

*Data are presented as medians with the 1st and 3rd quartiles in parentheses.

**Table 2:** Patient and tumor characteristics according to lateral cervical lymph node status in the training and validation cohorts.

	Training cohort (N = 400)		*p-*value	Validation cohort (N = 368)		*p-*value
	Lateral LN+ (n = 83)	Lateral LN- (n = 317)		Lateral LN+ (n = 100)	Lateral LN- (n = 268)	
Age (years)^a^	47 (33, 56)	45 (36, 54)	0.620	40 (31.25, 49.75)	47 (37, 54)	<0.001
Sex			0.491			
Female	69 (83.1%)	273 (86.1%)		74 (74.0%)	232 (86.6%)	0.004
Male	14 (16.9%)	44 (13.9%)		26 (26.0%)	36 (13.4%)	
Pathological tumor size (mm)^a^	20 (14, 25)	16 (13, 21)	0.011	17.50 (13, 25)	15 (12, 20)	0.001
Central LN metastasis			< 0.001			< 0.001
Negative	8 (9.6%)	162 (51.1%)		13 (13.0%)	149 (55.6%)	
Positive	75 (90.4%)	155 (48.9%)		87 (87.0%)	119 (44.4%)	
Rad-score*	-1.27 (-1.42, -0.97)	-1.48 (-1.64, -1.23)	< 0.001	-1.21 (-1.40, -1.00)	-1.36 (-1.56, -1.09)	< 0.001

*Data are presented as medians with the 1st and 3rd quartiles in parentheses.

### Feature selection and building the radiomics signature

**Fig 1:**
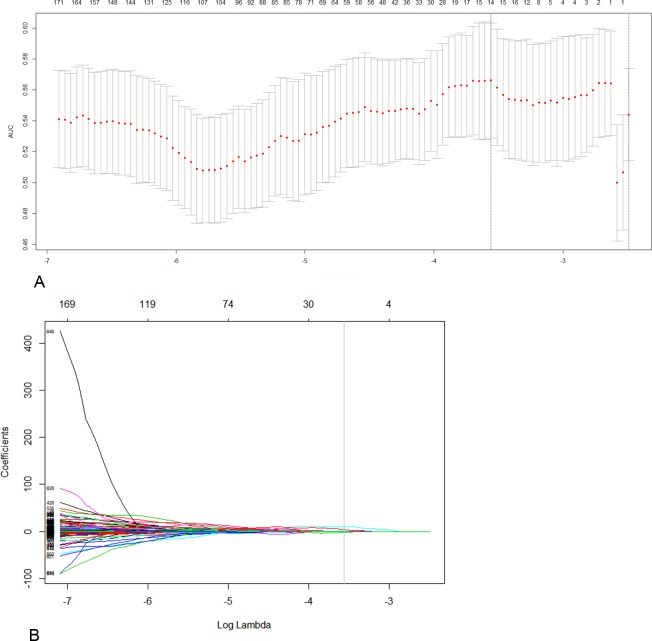
Imaging feature selection using the least absolute shrinkage and selection operator (LASSO) logistic regression model in the training cohort. (A) Tuning parameter (λ) in the LASSO model was selected under the minimum criteria. The area under the receiver operating characteristic curve (AUC) was plotted versus log (λ). Dotted vertical lines were drawn at the optimal values by using the minimum criteria and the 1 standard error (SE) of the minimum criteria (1-SE criteria) according to 10-fold cross validation. (B) LASSO coefficient profiles of the 730 features. A coefficient profile plot was produced against the log (λ) sequence. Vertical line was drawn at the value selected using 10-fold cross validation, where optimal λ resulted in 14 nonzero coefficients.

Based on the training cohort, 727 extracted features were reduced to 14 potential predictors using the LASSO regression model (Fig 1). These 14 features were included in the Rad-score calculation formula (S2 Appendix). The Rad-score of each nodule in the training and validation cohorts was calculated using this formula. Distribution of the Rad-score for patients with and without lateral LNM in the training and validation cohorts are displayed in Table 2. In both cohorts, the Rad-score was significantly different between patients with and without lateral LNM (all *p* < 0.001).

### Radiomics signature discrimination

**Fig 2:**
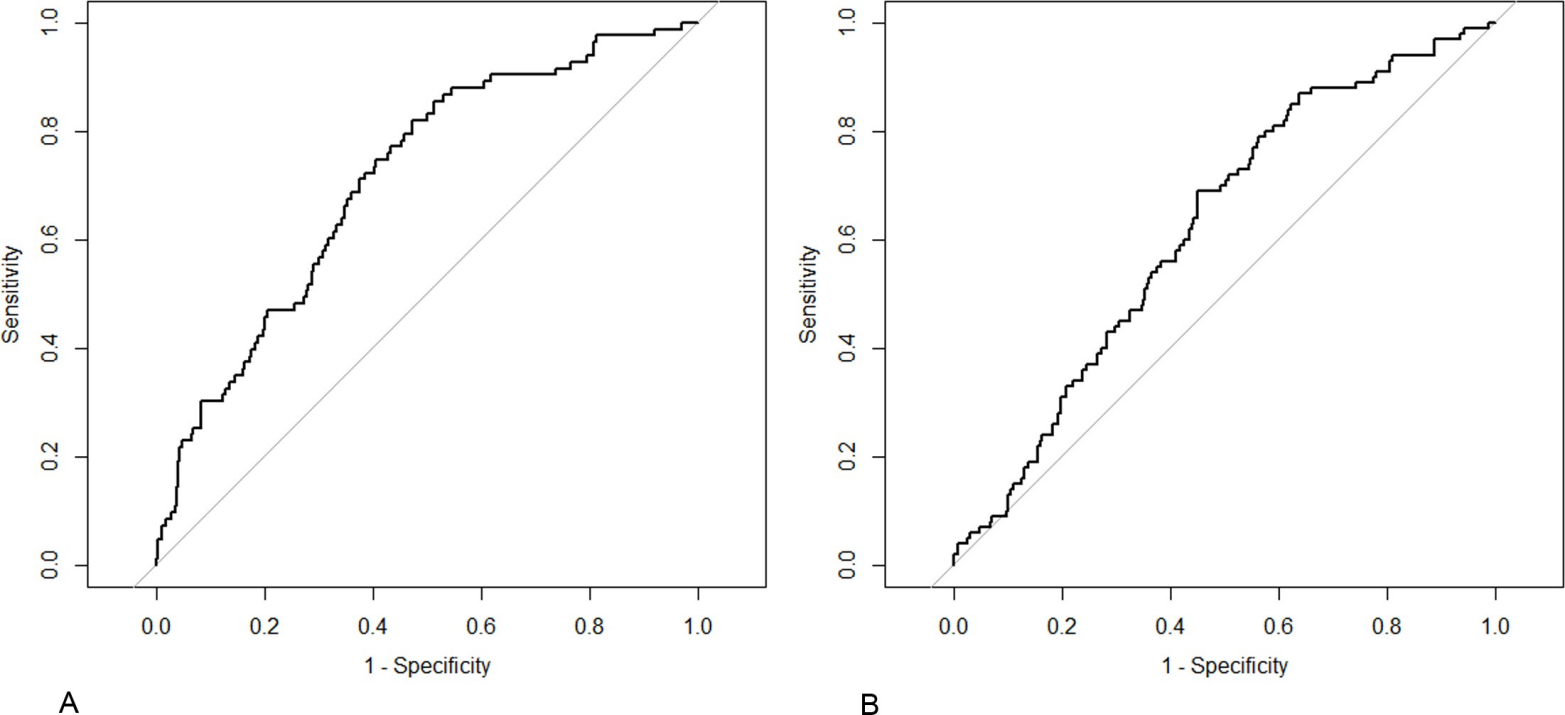
Receiver operating characteristic curves of the radiomics signature for predicting lateral cervical LNM. (A) Training cohort (AUC: 0.710 [95% CI: 0.649, 0.770]) and (B) validation cohort (AUC: 0.621 [95% CI: 0.560, 0.682]).

ROC curves of the radiomics signature were plotted to show the performance of the radiomics signature in predicting lateral cervical LNM in the training and validation cohorts (Fig 2). In the training cohort, the radiomics signature yielded an AUC of 0.710 (95% CI: 0.649, 0.770) for predicting lateral LNM. In the validation cohort, the radiomics signature yielded an AUC of 0.621 (95% CI: 0.560, 0.682)

## Discussion

We developed a radiomics signature as a new approach to preoperatively predict lateral cervical LNM in cPTC. Our study demonstrated that US radiomic features of the primary tumor were associated with lateral cervical LNM status and showed that the radiomics signature could potentially predict lateral LNM in patients with cPTC.

US features suggestive of metastatic LNs have been well established in literature, but no single US feature is adequately sensitive for the detection of cervical LNs with metastatic thyroid cancer [7]. Even when using a combination of suspicious US features, preoperative neck US is inevitably affected by interobserver variability. Evaluation results for lateral cervical lymph nodes vary the most among physicians, with the reported positive predictive values ranging from 38.5% to 64% according to different levels of experience [10]. Previous research has also focused on US or the pathologic features of the primary thyroid tumor itself, and has reported that a high suspicion US pattern, upper pole location, extrathyroidal extension, presence of calcifications, and central LNM are associated with lateral LNM [19–21]. However, such qualitative imaging features are also based on the judgment of the performing physician and therefore, prone to interobserver variability. In contrast, utilizing quantitative imaging features of the primary tumor enables an approach that is less affected by observer performance and thus, could be a more promising tool for clinical practice.

Radiomics has recently shown potential for achieving personalized medicine across a wide range of cancer types and has been reported to facilitate individualized prediction of LNM in patients with colorectal and bladder cancer [14,22]. However, its potential has been less investigated in thyroid cancer, and fewer or no studies have focused specifically on its association with lateral LNM. Although several previous studies have applied histogram and texture analysis in thyroid nodules, most have investigated only a small number of imaging parameters and were not based on high-dimensional data [23–28]. Furthermore, the majority of studies applying histogram or texture analysis have focused on differentiating malignant and benign thyroid nodules [23–27,29]. Therefore, in this study, we attempted to develop a radiomics signature for the prediction of lateral LNM in patients with cPTC based solely on the radiomic features of the primary thyroid tumor.

In two previous studies that investigated the association between B-mode US-based radiomic features of the thyroid tumor and cervical lymph node metastasis in patients with PTC, the radiomics model showed AUC values ranging from 0.727 to 0.81 [16,17]. These studies differ from ours in that lymph node metastasis including both central and lateral cervical lymph nodes was the investigated outcome, and a support vector machine classifier was employed to build the radiomics models. Interestingly, the performance of the radiomics model was higher in the study that used one US system without a separate validation set (AUC: 0.81) [16]. The other study collected images from three different US machines and tested the performance of the radiomics model in a separate testing cohort, resulting in an AUC value of 0.727, similar to our study [17]. It has been reported that radiomics models are dependent on the type of the US machine used, which may partly explain for the fair performance of the radiomics signature in our study [30]. In addition, our study showed slightly lower discriminative performance compared with the prior studies, possibly due to the different primary outcome and that only first order, textural, and waveleft-based features were utilized to build the radiomics signature. Other radiomic features which more directly reflect tumor shape, margin or tumor position may further improve its performance [17]. However, we found that among the 14 selected radiomic features, 10 were waveleft-based features, implying the importance of including higher-order statistical methods for feature extraction.

A previous meta-analysis reported that US showed an overall AUC of 0.85 in diagnosing lateral cervical LNM with a sensitivity of 75% (95% CI: 68–75) and a specificity of 97% (95% CI: 93–99) [9]. Similar results were obtained in a more recent study, in which US demonstrated a summary sensitivity of 71% (95% CI: 57–82) and a specificity of 85% (95% CI: 64–94) [31]. Despite its high accuracy, the performance of US is affected by the experience of the physician—among aspects of staging US, only the performance of diagnosing lateral LNM significantly differed between experienced and less experienced physicians [10]. Recently, a pilot study reported the development of a deep learning-based US computer-aided diagnostic system for the diagnosis of metastatic lymph nodes, and the diagnostic system showed an accuracy, sensitivity and specificity of 83.0%, 79.5% and 87.5%, respectively [32]. However, this would still require the observer to detect and image the lymph node for further analysis, and thus, results would likely be affected by interobserver variability. Although our approach allows evaluation to be relatively independent from physician experience, its diagnostic performance in the validation cohort was slightly lower than the training cohort, achieving an overall AUC of 0.621. As our study population consisted of patients imaged between 2004 and 2007, further improvements may be achieved in future studies which utilize images of even higher image resolution.

The limitations of our study include the retrospective nature of data collection and the lack of external validation for the model, as we used a validation cohort that was drawn from the same institution. Another limitation is that lateral compartment neck dissection was selectively performed in patients diagnosed with lateral LNM by preoperative fine-needle aspiration or intraoperative frozen biopsy. Considering that subclinical microscopic LNM occurs frequently in patients with PTC, the number of lateral LNM could have been underestimated. Finally, we utilized images obtained from several different US systems. Radiomic features have been reported to be affected by the type of US machine used for image acquisition, and this may have affected our results. However, our study results also show the potential of building and applying US-based radiomics signatures using multiple US systems, which could expand its potential for clinical application.

In conclusion, we developed a US radiomics signature based solely on imaging features of the primary tumor for the prediction of lateral cervical LNM in patients with cPTC. Although its discriminatory performance was slightly lower in the validation cohort, our study shows the potential of applying US radiomic features of the primary tumor alone for the prediction of lateral LNM. More studies are required for further validation and future improvement of radiomics-based preoperative prediction in patients with cPTC.

## Supporting information

S1 Appendix Radiomics feature extraction methodology. (DOCX)

S2 Appendix Radiomics score (Rad-score) calculation formula. (DOCX)

S1 File Study data set. (XLSX)
