# Supplementary material for: Radiomics signature for prediction of lateral lymph node metastasis in conventional papillary thyroid carcinoma
Source: PLoS One. 2020 Jan 15;15(1):e0227315. doi: 10.1371/journal.pone.0227315 (PMC6961896; doi:10.1371/journal.pone.0227315)
Supplement: S1 Appendix — (DOCX) [file pone.0227315.s001.docx]

**Title: Radiomics Signature for Prediction of Lateral Lymph Node Metastasis in Conventional Papillary Thyroid Carcinoma**

**S1 Appendix.** **Radiomics feature extraction methodology**

For feature selection, representative US images were chosen from images that were previously captured by the radiologist at the time of the examination and were retrieved from the picture archiving and communication system. A radiologist (V.Y.P.), who had 7 years of experience in thyroid US imaging, manually segmented the ROIs around the boundary of the index tumor on the representative US image. Afterwards, the position information of the ROI boundary was collected and applied to the US image with no ROI markings to extract the **
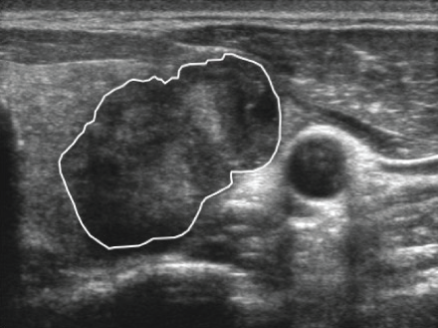

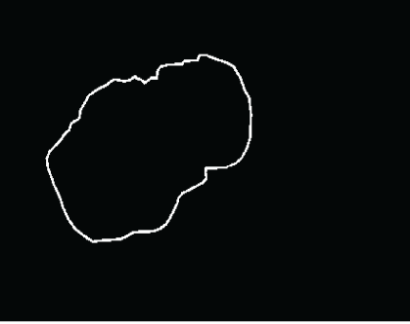

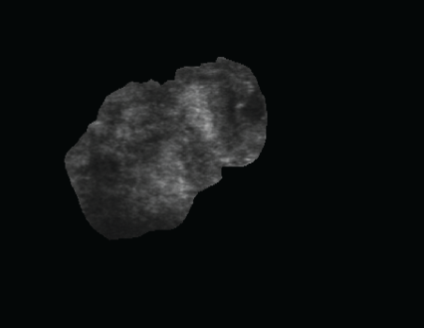
**ROI (see Fig. S1).

**(a) (b) (c)**

**Fig S1. (a)** ROI-segmented US image; **(b)** boundary of ROI; **(c)** ROI extraction from the original US image (before ROI marking)

First, the first order statistics values were collected such as energy, entropy, kurtosis, skewness, standard deviation, variance, maximum, minimum, median, mean, mean absolute deviation, range, root mean squares, and uniformity. In order to collect the textural features which describe patterns or the spatial relations with neighborhood pixels, the gray level co-occurrence matrix (GLCM) and gray level run-length matrix (GLRLM) were calculated after each extracted ROI image had been normalized for fair comparison (using min-max). The histogram of intensities of individual ROI images was calculated using ‘imhist’ function in MATLAB 2016b with 256 bins using a bin width of 1 (Fig. S2). The GLCM with distance 1 and GLRLM were calculated in angles 0, 45, 90, and 135 degrees. Then, the corresponding autocorrelation, cluster prominence, cluster shade, cluster tendency, contrast, correlation, difference entropy, dissimilarity, energy, energy (H), homogeneity 1, homegeneity 2, informational measure of correlation 1, informational measure of correlation 2, inverse difference moment normalized, inverse difference normalized, inverse variance, maximum probability, sum average, sum entropy, sum variance, variance, short run emphasis, long run emphasis, gray level non-uniformity, run length non-uniformity, run percentage, low gray level run emphasis, high gray level run emphasis, short run low gray level emphasis, short run high gray level emphasis, long run low gray level emphasis, and long run high gray level emphasis were obtained.

**Reference:** Aerts HJ, Velazquez ER, Leijenaar RT, Parmar C, Grossmann P, Carvalho S, et al. Decoding tumour phenotype by noninvasive imaging using a quantitative radiomics approach. Nat Commun. 2014;5: 4006. doi:10.1038/ncomms5006 PMID:24892406


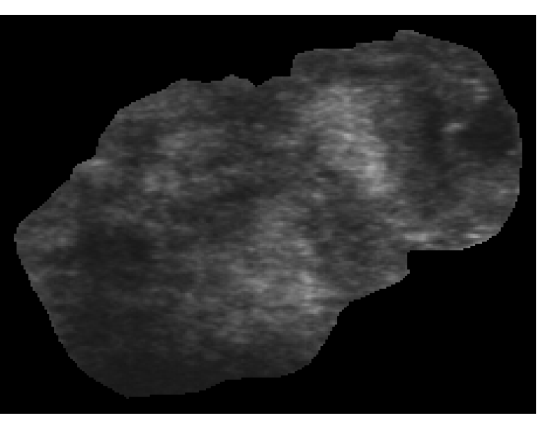

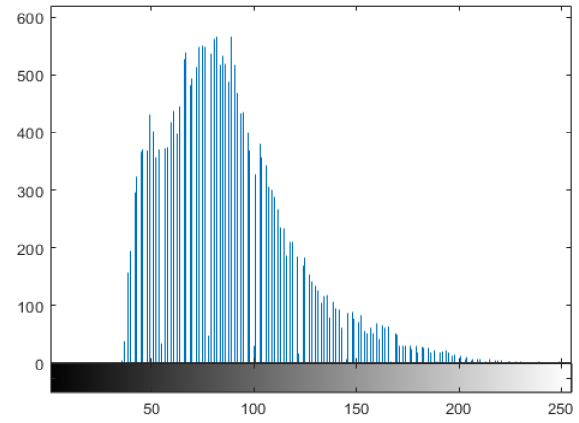


**(a) (b)**

**Fig S2. (a)** ROI; **(b)** Intensity histogram of ROI

For further feature extraction, we used the 2 dimensional discrete one-level wavelet transform to decompose the original US image using the ‘coiflet 1’ wavelet. The four discrete weavelet decompositions, HH, HL, LH, LL were utilized, where H and L are high- and low-pass filters in the x,y-directions, respectively (Fig. S3). All of the above first order statistics as well as GLCM, GLRLM-related feature values were calculated and used in feature analysis.


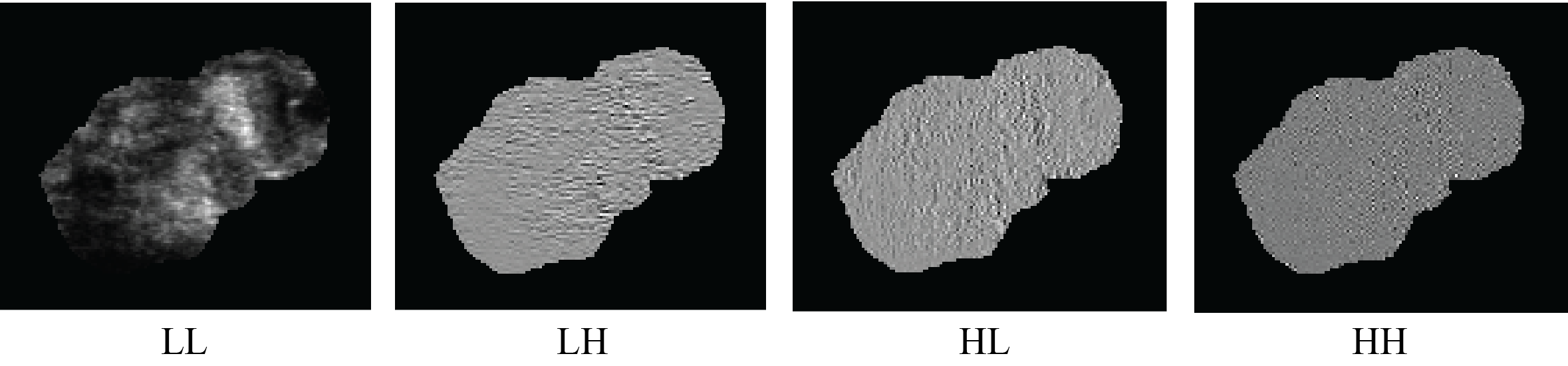


**Fig S3.** Wavelet decompositions, LL, LH, HL, and HH of the ROI image in **Fig S2 (a)**
