# Supplementary material for: Radiomics signature for prediction of lateral lymph node metastasis in conventional papillary thyroid carcinoma
Source: PLoS One. 2020 Jan 15;15(1):e0227315. doi: 10.1371/journal.pone.0227315 (PMC6961896; doi:10.1371/journal.pone.0227315)
Supplement: S2 Appendix — (DOCX) [file pone.0227315.s002.docx]

**Title: Radiomics Signature for Prediction of Lateral Lymph Node Metastasis in Conventional Papillary Thyroid Carcinoma**

**S2 Appendix. Radiomics score (Rad-score) calculation formula**

Rad score =

-10.7862433825983-0.0986752298619824$\times$se_45

+ 0.00145357315099227 $\times$lre_45 + 0.0028392037848861 $\times$lrlgle_45

+ 0.000980650829318763 $\times$lrlgle_135 + 0.0665714583895183$\times$HH_skew_0

+ 1.18011191420171$\times$HH_sre_135 - 1.94778026700035 $\times$HH_lglre_135

- 0.0729888993163001 $\times$ HL_kurt_0 + 10.1339169197946 $\times$ HL_idmn_90

+ 0.432336588225887 $\times$ HL_mp_135 + 0.000554433453755171$\times$LL_max_0

- 0.103128090052978$\times$LL_imc1_45- 0.115281385803744$\times$LL_de_135

+ 4.49211684595421$\times$LL_srlgle_135

where the values initiating with LL, LH, HL and HH are the quantities obtained from each wavelet subimages, and the values without them are from the original image, withhe numbers 0, 45, 90, 135 indicating the direction for which GLCM and GLRLM are calculated. The abbreviations in the above ‘Rad score’ are as follows.

First order statistics: Let **J** be the image matrix with *n* pixels and and let **P** be the first order histogram with 256 intensity levels. Then ‘mad=mean absolute deviation’, ‘med=median’ and ‘uni=uniformity’ are calculated as

$$\text{mad=}\frac{1}{n}\sum_{k=1}^{n} \left| \mathbf{J}\left( k \right)-\mathrm{mean}\left( \mathbf{J} \right) \right|, med=median of \mathbf{J,}\text{uni=}\sum_{k=1}^{256} \mathbf{P}\left( k \right)^{2}\mathbf{.}$$

Gray level co-occurrence matrix (GLCM): Let **M** be the gray level co-occurrence matrix adjacent to one pixel apart in the directions of 0, 45, 90 or 135. The number of levels is set to 10; that is, the values of **J** are rescaled as integers between 1 to 10, based on the minimum and maximum values of the given image intensity. Then, we have ‘de=difference entropy’, ‘idmn=inverse difference moment normalized’, ‘imc1=informational measure of correlation 1’, ‘imc2=informational measure of correlation 2’, ‘iv=inverse variance’ and ‘se=sum entropy’ with ‘ent(f)=the entropy of f’, $de=\sum_{k=0}^{9} m_{m}\left( k \right) \log_{2} m_{m}\left( k \right)$,

$$idmn=\sum_{i=1}^{10} \sum_{j=1}^{10} \frac{\mathbf{M}\left( i,j \right)}{1+\frac{\left( i-j \right)^{2}}{100}}, iv=\sum_{i=1}^{10} \sum_{j=1}^{10} \frac{\mathbf{M}\left( i,j \right)}{\left( i-j \right)^{2}}\left( i\neq j \right), imc1=\frac{\alpha-a1}{\max\{e\left( m_{x} \right),e\left( m_{y} \right)\}}, imc2=\sqrt{1-e^{-2(a2-\alpha)}}, se=-\sum_{k=2}^{20} m_{p}\left( k \right)\log_{2} m_{p}(k)$$

in which $m_{x}\left( k \right)=\sum_{j=1}^{10} \mathbf{M}(i,j),m_{y}\left( k \right)=\sum_{i=1}^{10} \mathbf{M}(i,j), \alpha=-\sum_{i,j=1}^{10} \mathbf{M}\left( i,j \right)\log_{2} \mathbf{M}\left( i,j \right)$,

$$m_{p}\left( k \right)=\sum_{i=1}^{10} \sum_{j=1}^{10} \mathbf{M}(i,j), \begin{matrix} i+j=k \\ k=1,\cdots,20 \end{matrix} , m_{m}\left( k \right)=\sum_{i=1}^{10} \sum_{j=1}^{10} \mathbf{M}(i,j),\begin{matrix} |i-j|=k \\ k=0,\cdots,9 \end{matrix} ,$$

and $a1=-\sum_{i,j=1}^{10} \mathbf{M}\left( i,j \right)\log m_{x}\left( i \right)m_{y}\left( j \right), a2=-\sum_{i,j=1}^{10} m_{x}\left( i \right)m_{y}(j)\log m_{x}\left( i \right)m_{y}\left( j \right)$.

Gray level run-length matrix (GLRLM)

: Let **G** be the gray level run-length matrix and it provides the size of homogeneous runs for each gray level (we set 16 gray levels) in the directions of 0, 45, 90, and 135. The parameters are ‘lglre = low gray level run emphasis’, ‘rp= run percentage’, ‘sre= short run emphasis’ and

‘srlgle = short run low gray level emphasis’ that are calculated as

$rp=\sum_{i=1}^{16} \sum_{j=1}^{N} \mathbf{G}(i,j)/n,$

$$lglre=\frac{\sum_{i=1}^{16} \sum_{j=1}^{N} \mathbf{G}(i,j)/i^{2}}{\sum_{i=1}^{16} \sum_{j=1}^{N} \mathbf{G}(i,j)},sre=\frac{\sum_{i=1}^{16} \sum_{j=1}^{N} \mathbf{G}(i,j)/j^{2}}{\sum_{i=1}^{16} \sum_{j=1}^{N} \mathbf{G}(i,j)},\mathrm{srlgle}=\frac{\sum_{i=1}^{16} \sum_{j=1}^{N} \mathbf{G}(i,j)/{(i}^{2}j^{2})}{\sum_{i=1}^{16} \sum_{j=1}^{N} \mathbf{G}(i,j)}$$

where N =max(r,c) with the rxc-quantized matrix of the given image (or image matrix **M**) using quantization level 16.
